# Bone metastases from breast cancer: associations between morphologic CT patterns and glycolytic activity on PET and bone scintigraphy as well as explorative search for influential factors

**DOI:** 10.1007/s12149-017-1202-3

**Published:** 2017-09-01

**Authors:** Tsutomu Sugihara, Mitsuru Koizumi, Masamichi Koyama, Takashi Terauchi, Naoya Gomi, Yoshinori Ito, Kiyohiko Hatake, Naohiro Sata

**Affiliations:** 1 Department of Breast Oncology, Yasugi Daiichi Hospital, 899-1 Yasugi, Yasugi, Shimane 692-011 Japan; 2 Department of Nuclear Medicine, Cancer Institute Hospital, 3-8-31 Ariake, Koto-ku, Tokyo, 135-8550 Japan; 3 Department of Diagnostic Radiology, Cancer Institute Hospital, 3-8-31 Ariake, Koto-ku, Tokyo, 135-8550 Japan; 4 Department of Breast Medical Oncology, Cancer Institute Hospital, 3-8-31 Ariake, Koto-ku, Tokyo, 135-8550 Japan; 5 Department of Hematology Oncology, Cancer Institute Hospital, 3-8-31 Ariake, Koto-ku, Tokyo, 135-8550 Japan; 6 Department of Gastrointestinal Surgery, Jichi Medical University, 3311-1 Yakushiji, Shimotsuke, Tochigi 329-0498 Japan

**Keywords:** Bone metastases, FDG-PET, Bone scintigraphy, Breast cancer

## Abstract

**Background:**

This study aimed to compare the detection of bone metastases from breast cancer on F-18 fluorodeoxyglucose positron emission tomography (FDG-PET) and bone scintigraphy (BS). An explorative search for factors influencing the sensitivity or uptake of BS and FDG-PET was also performed.

**Methods:**

Eighty-eight patients with bone metastases from breast cancer were eligible for this study. Histological confirmation of bone metastases was obtained in 31 patients. The bone metastases were visually classified into four types based on their computed tomography (CT) appearance: osteoblastic, osteolytic, mixed, and negative. The sensitivity of BS and FDG-PET were obtained regarding CT type, adjuvant therapy, and the primary tumor characteristics. The FDG maximum standardized uptake value (SUV_max_) was analyzed.

**Results:**

The sensitivities of the three modalities (CT, BS, and FDG-PET) were 77, 89, and 94%, respectively. The sensitivity of FDG-PET for the osteoblastic type (69%) was significantly lower than that for the other types (*P* < 0.001), and the sensitivity of BS for the negative type (70%) was significantly lower than that for the others. Regarding tumor characteristics, the sensitivity of FDG-PET significantly differed between nuclear grade (NG)1 and NG2–3 (*P* = 0.032). The SUV_max_ of the osteoblastic type was significantly lower than that of the other types (*P* = 0.009). The SUV_max_ of NG1 was also significantly lower than that of NG2–3 (*P* = 0.011). No significant difference in FDG uptake (SUV_max_) was detected between different histological types.

**Conclusion:**

Although FDG-PET is superior to BS for the detection of bone metastases from breast cancer, this technique has limitations in depicting osteoblastic bone metastases and NG1.

## Introduction

The bone is the most common site for distant metastases in patients with breast cancer, accounting for about 65% of patients with distant metastases and representing the first site of metastasis in 50% of patients [1, 2]. Conventional bone scintigraphy (BS) has been widely used to search for bone metastases, and it is undoubtedly useful because of its ability to evaluate the entire skeleton at a relatively low cost [3]. However, in some cases, it has low specificity and produces false-positive results due to uptake by benign lesions, such as osteoarthritis, fractures, and inflammation. Consequently, even experienced nuclear physicians often have difficulty in distinguishing bone metastases from benign disease [4, 5].

F-18 Fluorodeoxyglucose positron emission tomography (FDG-PET) is useful for staging cancer, detecting recurrences, and evaluating treatment effectiveness, and it is reportedly of particular value when searching for bone metastases from breast cancer [6, 7]. Multiple authors have concluded that FDG-PET/computed tomography (CT) is more sensitive for the detection of lytic bone metastases in patients with breast cancer, while BS is more sensitive for the detection of osteoblastic bone metastases [8–10]. A meta-analysis in 2008 showed no conclusive evidence regarding the superiority of BS or FDG-PET [11]. Since then, PET machines have been equipped with CT. A more recent meta-analysis in 2013 concluded that FDG-PET/CT is superior to BS in diagnosing bone metastases from breast cancer [12]. However, these previous studies did not discuss the tumor characteristics of breast cancer. In another study, non-FDG-avid osteoblastic bone metastases were more common in patients with invasive lobular carcinoma than in those with invasive ductal carcinoma [13].

In the present study, we further extended this investigation by directly comparing FDG-PET and BS for the detection of bone metastases from breast cancer. Each bone lesion was classified as the osteoblastic, osteolytic, mixed, or negative type based on its CT findings. We also performed an extensive search for factors influencing the sensitivity or uptake of BS and FDG-PET. We attempted to clarify the pitfalls of using FDG-PET for the detection of bone metastases.

## Patients and methods

### Patients

This single-institution retrospective study included consecutive patients with suspected bone metastases from histologically proven breast cancer treated at our hospital from February 2013 to December 2016. Patients who were suspected to have bone metastases on BS were pooled during this period, and those with definite bone metastases and FDG-PET/CT studies within 1 month were enrolled in this study. Bone involvement was histologically confirmed by biopsy, especially in cases of oligo bone metastases. If biopsy was difficult to perform or multiple bone lesions were present, the diagnosis was established clinically: confirmation was performed by other imaging modalities such as CT and magnetic resonance imaging, and by clinical follow-up.

The primary tumor characteristics as histological subtypes, tumor nuclear grades (NG), estrogen receptor (ER) status, and human epidermal growth factor receptor type 2 (HER2) status were recorded. We also investigated the effect of chemotherapy or hormone therapy on the diagnosis of bone metastases. The patients were divided into no treatment at the diagnosis of bone metastases, adjuvant hormone therapy, or adjuvant chemotherapy.

This study was done in accordance with ethical standard laid down in the 1964 Declaration of Helsinki and its later amendments, and this study was approved by our local ethical committee. Informed consent was waived for this type of study.

### BS, FDG-PET/CT, and CT

BS was performed approximately 3 h after an intravenous injection of 740 MBq technetium-99m methylene diphosphonate (^99m^Tc-MDP, Fujifilm RI Pharma Co. Ltd., Tokyo, Japan). Whole-body images were obtained using three different gamma cameras (ADAC Forte, Toshiba ECAM, and GE Infinia) equipped with low energy high-resolution parallel-hole collimators. The matrix size was 256 × 1024. The energy peak was centered at 140 keV with a 15% window. Scan speed of whole body was 20 cm per min.

Patients fasted for at least 6 h before being injected with 4 MBq/kg FDG and then whole-body image acquisition started at 60 min later from the top of the skull to the mid-thigh using an Aquiduo PET/CT scanner (Toshiba, Japan) or Discovery 600 PET/CT scanner (GE, USA). Emission data were acquired for 2–3 min per bed position. The PET images were reconstructed using an iterative algorithm (attenuation-weighted ordered subset expectation maximization: 4 iterations, 14 subsets) with an 8-mm Gaussian filter, a 128 × 128 matrix (3.9 mm/pixel) and 81 slices (2 mm/slice). Whole-body CT scanning proceeded under the following parameters: 120 kV; auto exposure control system (noise level: SD 10); 512 × 512 matrix; beam pitch, 0.94; 2 mm × 16-row mode. Maximum of standardized uptake value (SUV_max_) was measured from the representative bone metastatic lesion: biopsied site or representative site. Each patient had one SUV_max_.

CT studies were performed by a multi-detector GE-discovery 750 HD (64 rows, GE Healthcare Japan, Hino, Tokyo, Japan). CT scans were reconstructed with a 2-mm thickness at 5-mm intervals. Lesions of bone metastases on CT scan were visually classified by multi-slice CT based on the degree of osteoblastic and osteolytic change into four types: osteoblastic, osteolytic, mixed and negative (not detectable). FDG-PET, BS and CT images were evaluated independently by two nuclear medicine physicians. The discordant number was 2/88 in BS, 7/88 in CT, and 2/88 in FDG results. The discrepant cases were discussed by two nuclear physicians, and the final version was decided.

### Analysis and statistical methods

Step 1: The sensitivities of BS and FDG-PET were calculated with the CT four types of bone metastases on all patients. The contingency table analysis was performed using Fisher’s exact test.

Step 2: The sensitivity of BS and FDG-PET were calculated with respect to CT type, systemic therapy, and the primary tumor characteristics at the time of initial treatment. A contingency table analysis was performed using Fisher’s exact test.

Step 3: The FDG SUV_max_ of the bone metastases were compared with respect to factors showing a statistically significant difference in the above analysis and influential factors. Data were statistically analyzed using the Mann–Whitney *U* test.

Multivariate analysis was not applied because the number of patients was too small. Statistical analysis software (SPSS version 24; IBM Corp., Armonk, NY, USA) was used. A *P* value of *P* < 0.05 was considered to indicate statistical significance.

## Results

**Fig. 1:**
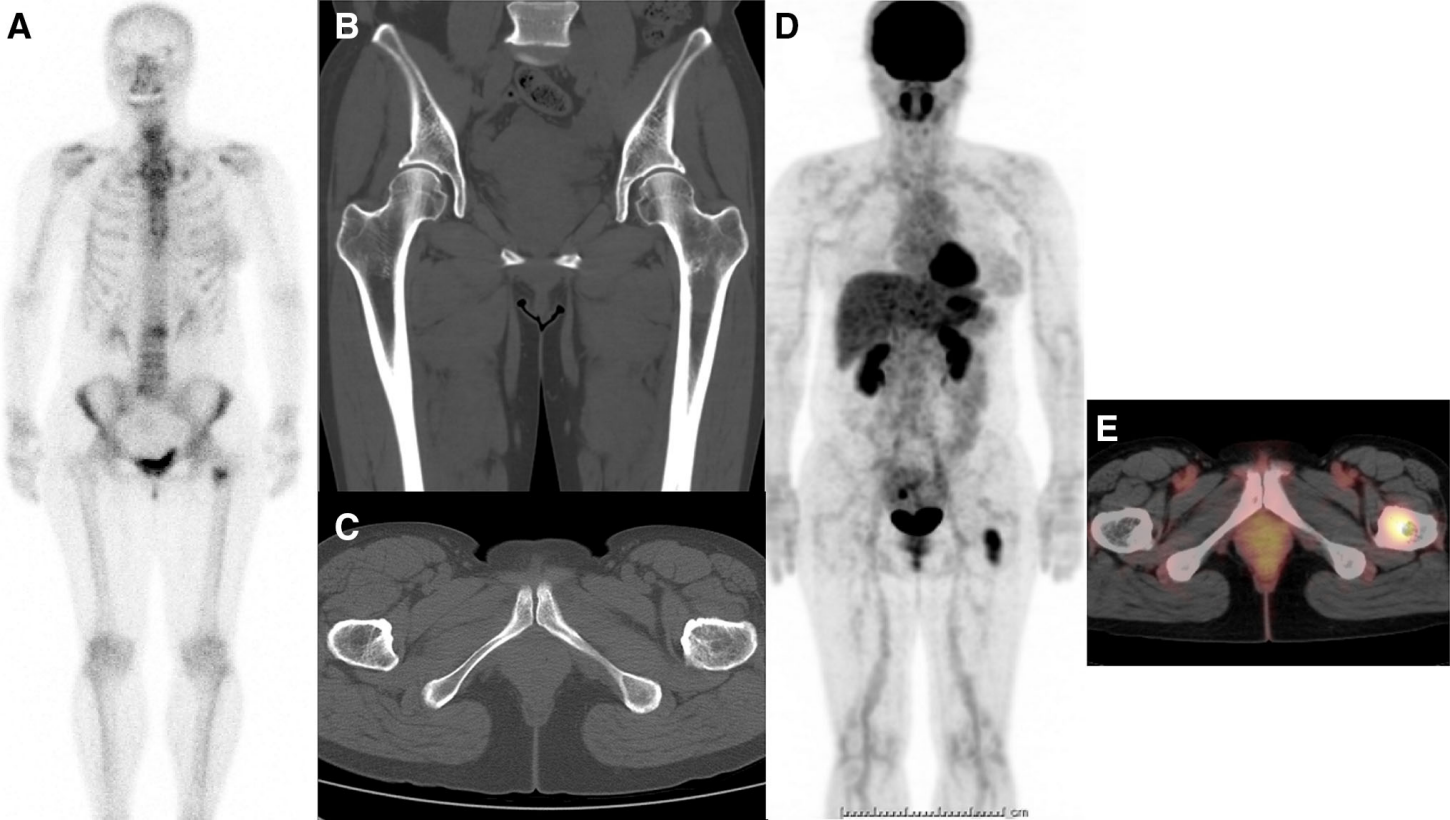
A 51-year-old female had right breast cancer surgery 10 years ago. She showed a tumor marker elevation (carcinoembryonic antigen), and bone scintigraphy (BS) was performed. A hot spot was shown in her left femur (around lesser trochanter) on BS (**a**). However, following CT could not demonstrate the lesion (**b** coronal, **c** axial). Therefore, FDG-PET/CT study was carried out, and an intense FDG uptake was noticed on her left femur (**d** whole-body FDG image, **e** fusion axial image). CT-guided bone biopsy revealed the lesion as metastasis pathologically

Suspected bone metastases from breast cancer were present in 149 patients, 88 of whom had definite bone metastases and had undergone both BS and FDG-PET studies within 1 month. The median patient age at diagnosis was 60 years (range 31–85 years). Thirty-five of the 88 patients underwent biopsy or surgery for the suspected bone metastases, and histological confirmation was obtained in 31 patients. The other 57 patients were clinically diagnosed with bone metastases by their imaging findings and clinical course. Bone metastases were classified according to their CT findings as osteoblastic in 16 patients, osteolytic in 31, mixed in 21, and negative in 20. The final diagnosis of CT-negative patients was made as follows: 6 patients were confirmed by bone biopsy, 4 by MRI, and 10 by later studies (CT appearance turned to be evident). Figure 1 shows a CT-negative patient. A hot spot was shown in the left femur (around the lesser trochanter) on BS; however, CT study did not detect any abnormality (CT negative). Therefore, FDG-PET/CT study was carried out to evaluate the left femur lesion, and intense FDG uptake was detected. Then, CT-guided biopsy confirmed the lesion as metastasis pathologically.

The patients’ demographic characteristics are shown in Table 1.

**Table 1:** Patients’ characteristics

Histology confirmed (bone metastasis)	
Yes	31^a^
No	57
Histology	
Invasive ductal cancer	76
Invasive lobular cancer	7
Others	5
Nuclear grade	
1	17
2	32
3	14
Unknown	25
Estrogen receptor	
Positive	77
Negative	11
HER 2^b^	
Positive	51
Negative	37
Systemic adjuvant therapy	
No	49
Hormone	37
Chemotherapy	2

^a^Number of patients

^b^Human epidermal growth factor receptor type 2

The sensitivities of the three imaging modalities (CT, BS, and FDG-PET) were 77% (68/88), 89% (78/88), and 94% (83/88), respectively. The sensitivities of BS and FDG-PET for the four CT-based types are shown in Table 2. The sensitivity of BS was 94% (15/16) for the osteoblastic type, 90% (28/31) for osteolytic, 100% (21/21) for mixed, and 70% (14/20) for negative. The sensitivity of FDG-PET was 69% (11/16) for the osteoblastic type, and 100% for the other three types. The sensitivity of FDG-PET for the osteoblastic type was significantly lower than that for the other types (*P* < 0.001). The sensitivity of BS for the negative type (70%, 14/20) was significantly lower than that for the other types (*P* = 0.008). There was no significant difference in the sensitivities of BS and FDG-PET with respect to the histological type, ER status, HER2 status, or systemic adjuvant therapy. A significant difference in the sensitivity of FDG-PET was observed between NG1 and NG2–3 (*P* = 0.032).

**Table 2:** BS and FDG-PET sensitivity for bone metastasis by CT type

CT types	BS	FDG
Osteoblastic	94% (15/16)	69% (11/16)
Osteolytic	90% (28/31)	100% (31/31)
Mixed	100% (21/21)	100% (21/21)
Negative	70% (14/20)	100% (20/20)
Total	89% (78/88)	94% (83/88)

Numbers in parentheses indicate (positive patient number/patient number)

Total CT sensitivity: 77% (68/88)

*BS* bone scintigraphy, *FDG* F-18 fluorodeoxyglucose, *CT* computed tomography

The patients’ SUV_max_ are shown in Table 3. The SUV_max_ was available in 77 of 88 patients. The CT type (osteoblastic vs. others) and NG (NG1 vs. NG 2–3) were chosen because they showed statistically significant differences in the preceding analysis (contingency table analysis). The histological type (invasive ductal cancer vs. invasive lobular cancer) was also included because it was one of the main investigation themes of this study. The SUV_max_ of the osteoblastic type was significantly lower than that of the other types (*P* = 0.009). The SUV_max_ of NG1 was significantly lower than that of NG2–3 (*P* = 0011). The median SUV_max_ of invasive lobular cancer was lower than that of invasive ductal cancer (median, 4.5 and 6.7, respectively); however, these values were not statistically different (*P* = 0.103) (Table 4).

**Table 3:** CT types, breast tumor data, and adjuvant therapy vs. BS and FDG sensitivities

	BS			FDG		
	Positive	Negative	*P*	Positive	Negative	*P*
Osteoblastic vs. others (CT)						
Osteoblastic	15^a^	1	0.682	11	5	**<0.01**
Others	63	9		72	0	
Invisible vs. others (CT)						
Negative	14	6	**0.008**	20	0	0.266
Others	64	4		63	5	
Histology						
Invasive ductal cancer	69	7	0.367	72	4	0.449
Invasive lobular cancer	5	2		7	0	
Others	4	1		4	1	
ER						
Negative	10	1	0.636	10	1	0.496
Positive	68	9		73	4	
HER 2						
Negative	32	5	0.415	35	35	0.65
Positive	46	5		48	48	
NG						
1	13	4	0.22	14	3	**0.032**
2	30	2		32	0	
3	12	2		13	1	
Systemic adjuvant therapy						
No	45	4	0.463	46	3	1
Hormone	31	6		35	2	
Chemotherapy	2	0		2	0	

^a^Number of patients

*BS* bone scintigraphy, *FDG* F-18 fluorodeoxyglucose, *CT* computed tomography, *ER* estrogen receptor, *HER2* human epidermal growth factor receptor type 2, *NG* nuclear grade

**Table 4:** FDG SUV_max_ by CT type, histology, and nuclear grade

	*n*	Median	Range	*P*
CT type				
Osteoblastic	14	4.85	1.5–13.7	**0.009**
Others	63	7.1	2.3–28.1	
Histology				
Invasive ductal cancer	66	6.7	1.5–28.1	0.103
Invasive lobular cancer	7	4.5	2.8–14.1	
Nuclear grade				
1	13	5	2–19.4	**0.011**
2 and 3	41	8.8	1.5–28.1	

FDG SUV_max_ data were available in 77 of 88 patients

*FDG* F-18 fluorodeoxyglucose, *SUV*_*max*_ maximum standardized uptake value, *CT* computed tomography

## Discussion

The diagnostic accuracy of FDG-PET and BS for bone metastases in patients with breast cancer has been exclusively compared and studied, but whether FDG-PET or BS is superior in detecting bone metastases remained inconclusive in a 2008 meta-analysis [11]. However, in a 2013 meta-analysis, FDG PET/CT had higher sensitivity and accuracy than BS for detection of bone metastases in patients with breast cancer [12]. Earlier reports suggested that FDG-PET, while highly sensitive for detecting osteolytic type bone metastases as shown on CT, had a lower detection ratio for osteoblastic-type metastases [8–10, 14]. The decreased FDG uptake in osteoblastic bone metastases might be explained by the following scenario: osteoblastic proliferation in osteoblastic metastases results in an increased bone matrix and relatively decreased cell density; this leads to lower FDG accumulation because FDG uptake in tissue reflects the underlying glucose metabolism and cell density [14]. In the present study, the SUV_max_ and sensitivity of FDG-PET for CT osteoblastic-type metastases were significantly lower than those for other CT types. Our study also showed that the sensitivity of BS was low for the CT-negative type. PET machines have recently been equipped with CT, and the FDG PET/CT results can be comprehensively evaluated using both FDG accumulation and CT features. Therefore, FDG-PET/CT allowed for detection of all bone metastases in our study.

Another factor that might influence on FDG uptake is histological differences. Previous studies have suggested that the FDG avidity of primary breast cancer is lower in patients with invasive lobular carcinoma than invasive ductal carcinoma [15–18]. The lower FDG avidity of invasive lobular carcinoma could be explained by the lower cellular density, proliferation rate, and number of GLUT glucose transporters in this breast cancer histology than in more common histologies [16, 18]. However, few studies have investigated the relationship between breast cancer histology and FDG avidity in patients with bone metastases. Dashevsky et al. [13] compared the histology of breast cancer and the FDG avidity in bone metastases from breast cancer. They reported that non-FDG-avid sclerotic osseous metastases were more common in invasive lobular cancer than in invasive ductal cancer. The present study results are not in agreement with their findings. All bone metastases in our patients with invasive lobular cancer were FDG avid (7/7). Of the seven patients, three patients were the CT-negative type and the other four were the CT-osteolytic type. The median FDG SUV_max_ of invasive lobular cancer was lower, but not statistically significant; the median FDG SUV_max_ of invasive lobular and ductal cancers were 4.5 and 6.7, respectively, but there was no statistically significant difference (*P* = 0.103). The reason for this discrepancy might be the small patient number in our study (*n* = 7). Another factor that differed between the present study and that by Dashevsky et al. [13] is that our bone metastases were newly diagnosed, but some patients received adjuvant therapy. Among seven patients with invasive lobular cancer, five did not receive adjuvant therapy, but two were diagnosed with bone metastases during adjuvant hormone therapy. Further investigation is needed to clarify the relationship between the histology of breast cancer and FDG avidity in bone metastases.

We identified a relationship between the NG of breast cancer and FDG avidity. Specifically, FDG avidity (both sensitivity and SUV_max_) of bone metastases in patients with NG1 cancer was significantly lower than that in patients with NG2–3 cancer. In other words, FDG uptake in bone metastases from primary tumors with a mild NG was lower than that from primary tumors with a more aggressive NG. This finding is unique but needs to be clarified in other studies because the number of patients was not enough in the present study. We found no significant correlation between the NG grade and CT type (data were not shown).

The limitations of the present study are its retrospective design and rather small number of patients, especially in the subgroups. The small number of patients prevented us from performing a multivariate analysis. Another limitation of the present study was that 31 of 88 patients were confirmed histologically as having bone metastasis, which means 57 patients were diagnosed clinically. We experienced difficulty in clinical diagnosis of bone metastasis in CT-negative (intertrabecular type) patients. There were 20 patients with CT-negative results. The final diagnosis of CT-negative patients was made as follows: 6 patients were confirmed by bone biopsy, 4 by MRI and follow-up, and 10 by later studies (CT appearance turned to be evident).

The development of single photon emission computed tomography with CT (SPECT/CT) made the planar bone scintigraphy as an old modality, although bone scan is still actively used in clinical practice. The computer-aided diagnosis such as Bone Navi made the old modality (bone scintigraphy) as a modern diagnostic tool. We hoped to conduct the study using bone SPECT/CT, however, bone SPECT/CT was not performed in any patients. This prevents us from performing the study using bone SPECT/CT.

In conclusion, the diagnostic performance of BS and FDG-PET was re-evaluated in patients with bone metastases from breast cancer, and histological confirmation of bone metastases was obtained in 31 of 88 patients. We considered that CT morphological type was an important factor for the diagnostic performance. The negative type often showed negative BS results, and the osteoblastic type often showed negative FDG-PET results. FDG-PET/CT has potential to serve as an excellent imaging modality because both FDG and CT data can be obtained in a single study, and each imaging modality compensates for the other’s weak points. We further performed the analysis of various factors influencing FDG accumulation. Significantly low FDG uptake was seen in osteoblastic-type bone metastases and bone metastases from primary breast cancer with a low NG. Other factors including the histological type showed no significant difference. These results would be of use when interpreting bone metastases.
